# Presence of Ebola virus in breast milk and risk of mother‐to‐child transmission: synthesis of evidence

**DOI:** 10.1111/nyas.14519

**Published:** 2020-10-28

**Authors:** Melisa Medina‐Rivera, Elizabeth Centeno‐Tablante, Julia L. Finkelstein, Pura Rayco‐Solon, Juan Pablo Peña‐Rosas, Maria N. Garcia‐Casal, Lisa Rogers, Pratiwi Ridwan, Sabrina Sales Martinez, Joyce Andrade, Alexander J. Layden, Juan Chang, Mildred P. Zambrano, Kate Ghezzi‐Kopel, Saurabh Mehta

**Affiliations:** ^1^ Division of Nutritional Sciences Cornell University Ithaca New York; ^2^ Department of Maternal, Newborn, Child and Adolescent Health and Ageing World Health Organization Geneva Switzerland; ^3^ Department of Nutrition and Food Safety World Health Organization Geneva Switzerland; ^4^ Department of Dietetics and Nutrition, Robert Stempel College of Public Health and Social Work Florida International University Miami Florida; ^5^ Hospital de Niños Roberto Gilbert Elizalde Guayaquil Ecuador; ^6^ Department of Epidemiology University of Pittsburgh Pittsburgh Pennsylvania; ^7^ Albert R. Mann Library Cornell University Ithaca New York

**Keywords:** Ebola virus, vertical transmission, perinatal transmission, mother‐to‐child transmission, breast milk, breastfeeding

## Abstract

To help inform global guidelines on infant feeding, this systematic review synthesizes evidence related to the presence of the Ebola virus (EBOV) in breast milk and its potential risk of viral transmission to the infant when breastfeeding. We relied on a comprehensive search strategy to identify studies including women with suspected, probable, or confirmed EBOV infection, intending to breastfeed or give breast milk to an infant. Our search identified 10,454 records, and after deduplication and screening, we assessed 148 full texts. We included eight studies reporting on 10 breastfeeding mothers and their children (one mother with twins), who provided breast milk samples for assessment. EBOV was detected via RT‐PCR or viral culture in seven out of ten breast milk samples. Four out of the five‐breastfed infants with EBOV‐positive breast milk were found positive for EBOV infection, and all of these EBOV‐positive infants died. Since previous reports have detected EBOV in tears, saliva, sweat, and contaminated surfaces, with the current evidence, it is not possible to conclude with certainty that breast milk was the main route of EBOV transmission.

## Graphical abstract

Understanding maternal‐to‐child transmission routes is key to lowering, and ultimately preventing, the exposure of Ebola virus (EBOV) in pediatric populations. We undertook a systematic review of the available scientific literature to determine whether EBOV can be transmitted through breast milk and to describe the outcomes of the infants that ingested EBOV laboratory‐confirmed breast milk.

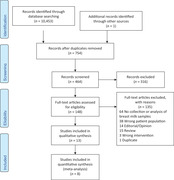

## Introduction

*Zaire ebolavirus*, the species of Ebola virus (EBOV) responsible for current outbreaks, belongs to the *Filoviridae* family of viruses, single‐strand ribonucleic acid pathogens known for causing virulent hemorrhagic fever in humans and other primate species.^1^, ^2^, ^3^, ^4^ The initial zoonotic transfer to a human host is predicted to occur through hunting, handling, or consuming infected forest animals.^5^, ^6^, ^7^, ^8^, ^9^ To this date, however, no clear natural host has been confirmed. Scientific reports suggest that a variety of bats, duikers, and other nonhuman primates are either natural viral reservoirs or play key epidemiological roles in the spillover and spread of EBOV.^5^, ^6^, ^7^, ^10^, ^11^, ^12^, ^13^, ^14^, ^15^ Following an index case, EBOV infection spreads primarily from human to human through direct contact with infected bodily fluids or contaminated fomites.^6^, ^16^, ^17^ Following an incubation period of 2–21 days, EBOV‐infected individuals develop nonspecific viral symptoms, such as fatigue, anorexia, fever, and malaise.^8^, ^18^, ^19^ As the infection progresses, clinical manifestations might also include gastrointestinal symptoms (abdominal pain, diarrhea, and vomit) that without proper care can lead to viral septic shock, multisystem organ failure, and ultimately death.^8^, ^19^, ^20^ Moreover, EBOV survivors can suffer from sequelae of infection for a year or longer, which includes debilitating or incapacitating musculoskeletal, neurological, auditory, visual, and gastrointestinal symptoms.^21^, ^22^, ^23^, ^24^, ^25^, ^26^

Since no specific antiviral drugs are licensed to treat EBOV infection in humans, management efforts are focused on providing supportive care.^27^, ^28^ Preventive measures, such as vaccines, have been developed and tested in human and nonhuman trials.^3^, ^29^, ^30^, ^31^, ^32^, ^33^ Most recently, the recombinant vesicular stomatitis virus–Zaire Ebola virus (rVSV‐ZEBOV) vaccine, developed by Merck & Co, acquired approval by the U.S. Food and Drug Administration for its robust margin of success during human clinical trials and a ring vaccination strategy to contain the spread among healthcare workers, EBOV‐positive contacts, and contacts of contacts during recent outbreaks.^32^, ^33^, ^34^, ^35^, ^36^ But uncertainty regarding the long‐term safety and immunogenicity of the vaccine remains, and even if enduring efforts to fill out these gaps are underway,^31^, ^37^ large‐scale implementation of routine vaccination might still be years or even decades away. Therefore, efforts rely on other Ebola control measures focused on reducing the transmission risk among susceptible communities. These control measures incorporate a package of interventions, namely, case management, infection prevention, and control practices, surveillance and contact tracing, proficient laboratory service, safe and dignified burials, and community engagement.^4^

Despite significant advances in research, management, and control efforts that have been put in place, EBOV infection persists as a public concern and health threat to susceptible populations, such as pregnant women and young children.^38^, ^39^ Particularly, EBOV infection during pregnancy is associated with fatal obstetrical and neonatal complications that include bleeding, miscarriage, stillbirth, and preterm delivery.^8^, ^40^, ^41^, ^42^, ^43^, ^44^, ^45^, ^46^, ^47^ While reports of mother‐to‐child EBOV transmission are limited, EBOV is suspected to transfer via transplacental, transvaginal, or breastfeeding routes. Evidence supporting this claim are studies confirming the presence of EBOV in maternal fluids, such as blood, vagina secretions, amniotic fluid, placenta, sweat, tears, urine, saliva, and breast milk, acquired during maternal acute and convalescent states of EBOV disease.^16^, ^17^, ^42^, ^43^, ^44^, ^47^, ^48^ Regardless of the route of vertical transmission, except for a few cases,^49^, ^50^ exposure to EBOV in neonatal populations is associated with high mortality rates.^8^, ^41^, ^42^, ^51^, ^52^ Modeled estimates in the 2015–2016 EBOV outbreak in the Bombali District in Sierra Leone suggest that 73% of children under 5 years exposed to EBOV died.^53^ This number, however, might be an underestimate mainly because of limited surveillance and underreporting in geographical locations with high EBOV incidence.^54^

Understanding maternal‐to‐child transmission routes is key to lowering, and ultimately preventing, the exposure of EBOV in pediatric populations. We undertook a systematic review of the available scientific literature to determine whether EBOV can be transmitted through breast milk and to describe the outcomes of the infants who ingested EBOV laboratory‐confirmed breast milk. Given the assessment and analysis of this evidence, the World Health Organization (WHO) has recommended that breastfeeding of a child should stop if Ebola virus infection is confirmed in a lactating woman or a breastfed child.^55^ As the Ebola outbreaks continue in some countries, it is important to maintain data surveillance and evidence‐informed guidelines for infant feeding to better support mother‐and‐child populations.

## Methods

A search strategy and review protocol were developed. The search was performed without any date or language restrictions. All electronic databases were last searched on July 21, 2020. Pertinent studies were identified as per the inclusion criteria.

### Inclusion criteria

#### Study design

We included all types of study designs available: randomized control trials, quasi‐randomized control trials, interrupted time‐series, prospective cohorts, retrospective cohorts, case studies, cross‐sectional studies, and surveillance reports.

#### Participants

Women with a suspected, probable, or confirmed case of EBOV infection at any time during pregnancy or postpartum, currently giving or intending to breastfeed or give expressed milk to an infant.

#### Exposure

Healthy infants and young children (≤2 years of age) consuming breast milk directly from the breast or expressed breast milk from a woman with suspected, probable, or confirmed EBOV infection.

#### Outcomes

The primary outcome included the identification of EBOV or associated viral molecules in breast milk, blood, sweat, or saliva. Various diagnostic methods are commonly utilized for the detection and identification of EBOV in bodily fluids. These methods include viral isolation by culturing, reverse transcription‐polymerase chain reaction (RT‐PCR) to detect viral nucleic acids, and enzyme‐linked immunosorbent assay (ELISA) to capture viral antigens or anti‐EBOV antibodies. Secondary outcomes included the infection of an infant with suspected, probable, or confirmed EBOV infection within 30 days of breastfeeding or receiving breast milk from a woman with suspected, probable, or confirmed EBOV infection.

### Search strategy

Key medical subject headings (MeSH) and term words related to EBOV (e.g., Ebola, Ebolavirus, and hemorrhagic fever), breastfeeding (e.g., breast milk, colostrum, and lactation), and the population of interest (e.g., pregnant women, pregnancy, and infant) were employed to conduct a thorough search on regional and international databases. The search included the following databases: Cochrane Central Register of Controlled Trials (CENTRAL), MEDLINE (PubMed), EMBASE, CINAHL, Web of Science (SSCI, SCI), BIOSIS, SCIELO, Global Index Medicus‐AFRO, EMRO, LILACS, PAHO, WHOLIS, WPRO, IMSEAR, IndMED, and Native Health Research Database. A detailed systematic search description can be found in the Supplementary Materials (online only).

### Study selection

The identified studies were imported into Covidence systematic review software (Veritas Health Innovation, Melbourne, Australia). Duplicates were removed. The titles and abstracts of the remaining studies were screened independently for eligibility by two review authors. Following title and abstract screening, full‐text articles were obtained from the identified studies and were further screened against the inclusion criteria. A third review author helped to solve disagreements for study inclusion during abstract and full‐text screening.

### Data extraction and management

Using a structured data collection form, two authors independently collected data from the selected studies. Any discrepancies that were encountered were resolved by discussion among reviewers. The data collected from the articles included information about the study design, setting, participants (number and characteristics), the interventions, and measured outcomes.

### Assessing the certainty of evidence

Two independent authors assessed the certainty of evidence from each study following the instructions outlined by the Grading of Recommendations, Assessment, Development, and Evaluation (GRADE) Working Group, GRADEprofiler software.^56^ The GRADE approach rates the evidence of certainty for each outcome as “high certainty” and can be downgraded to “moderate,” “low,” and “very low” on the basis of the study limitations (e.g., risk of bias, imprecision, inconsistency, indirectness, and publication bias). Any found conflicts with the ratings were resolved by discussion among the authors. The quality assessment report is presented as a narrative.

## Results

### Study designs

The literature search retrieved 10,453 records, and one record was identified through external sources. After removing duplicates, a total of 754 records were imported into Covidence. Following a second round of deduplication performed by the systematic review software, 464 studies were screened on the basis of their titles and abstracts. Studies were excluded if they were review articles, editorials, or had an *in vitro* focus on human milk composition and bioactivity. Studies were also excluded if breast milk samples were not collected, measured for infectious pathogens, spiked with EBOV under laboratory conditions, or belonged to nonhuman mammal species. Additional reasons for exclusion included studies of women coinfected with human immunodeficiency virus (HIV) or with acquired immunodeficiency syndrome.

**Figure 1:**
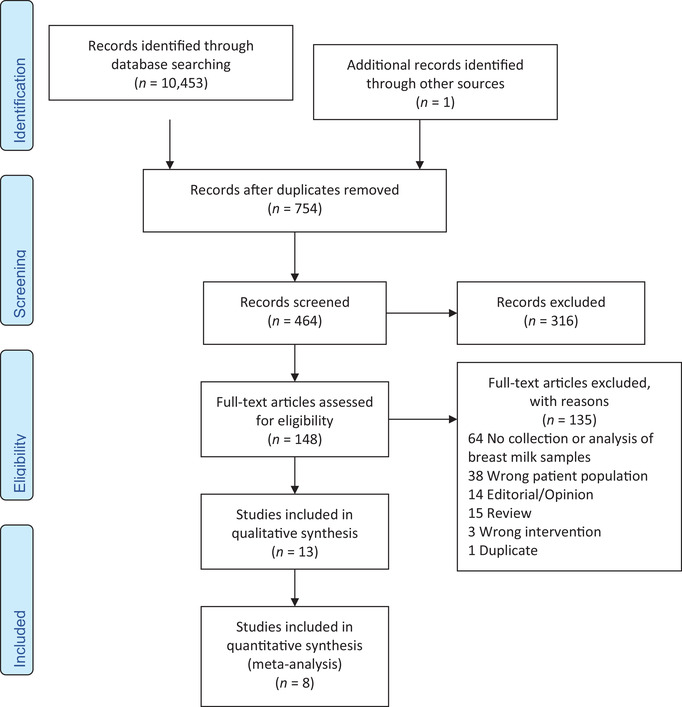
Prisma flow diagram.

Following title and abstract screening, a total of 148 articles were identified for full‐text eligibility against the inclusion criteria. We found a total of 13 records for data extraction and descriptive analysis. Among these, four studies were case reports,^57^, ^58^, ^59^, ^60^ three records reported on outbreak surveillance efforts,^16^, ^61^, ^62^ three records reported on a prospective observational study of the same cohort of EBOV disease survivors,^26^, ^63^, ^64^ one study was a methodology report of a cohort,^65^ and two records were conference abstracts.^66^, ^67^ For the methodology report and conference abstracts,^65^, ^66^, ^67^ no details of the population of interest or collected breast milk samples were found. Thus, these two reports are not included in the analysis. Three studies reported outcomes of the same prospective cohort (PostEbogui) study of EBOV disease survivors.^26^, ^63^, ^64^ We considered the breast milk findings from this cohort to correspond to the same samples in all articles and thus, we only describe the findings reported by Keita and collaborators.^63^ A PRISMA flow diagram of the systematic review search data is presented in Figure 1.

### Settings

The eight records informing the analysis of this review are from Uganda (*n* = 1),^16^ Sierra Leone (*n* = 1),^61^ Guinea (*n* = 4),^57^, ^58^, ^59^ United States (*n* = 1),^60^ and Liberia (*n* = 1).^62^

### Participants

The reports included a total of 11 women with a suspected or laboratory‐confirmed EBOV infection and their children (one mother with twins). Among these women, 10 mothers provided breast milk samples for laboratory assessment and, therefore, only they are accounted for in the analysis. EBOV maternal status was assessed by analyzing urine or blood specimens via molecular, serological, or viral culturing techniques. In two studies,^16^, ^63^ breastfeeding participants (*n* = 4) were reported to be positive for EBOV disease, although the laboratory methods to confirm maternal status were not described, two of these women were EBOV disease survivors.^63^ Of the remaining women, two out of six mothers were found to be EBOV positive by RT‐PCR,^57^, ^58^, ^59^, ^60^, ^61^, ^62^ and three women were found to have anti‐EBOV antibodies via ELISA.^59^, ^60^, ^62^ Of the cases that included information of antibody titers, one woman with records of a positive EBOV RT‐PCR test performed 13 months before birth was found to have IgG but no IgM antibodies before delivery.^60^ By contrast, the two other women both had negative RT‐PCR tests, performed at 2 and 9 months after delivery, and were deemed positive after antibody assessment showed the presence of EBOV‐specific antibodies in blood samples.^59^, ^62^ In one case,^62^ the woman was both IgG and IgM positive, whereas in the second case,^59^ the mother was IgG positive and IgM negative.

Of the six women whose clinical manifestations were reported, three were reported as asymptomatic, one showed symptoms of acute disease, and two were in the convalescent phase of EBOV disease at the time of sample collection. A total of 10 breast milk samples were collected from different women with confirmed or suspected EBOV disease. Nine of the breast milk samples were assessed by RT‐PCR and one by viral culturing. Seven out of 10 breast milk specimens were confirmed to be positive to EBOV.^16^, ^58^, ^59^, ^61^, ^63^ In one of the studies with positive breast milk samples,^63^ 168 breast milk samples from 109 different EBOV survivors were analyzed. Only two breast milk specimens belonging to different women were positive. In one case, six breast milk specimens were collected from the same woman. The first sample, collected at 500 days after being discharged from the Ebola treatment center, was positive. Five subsequent samples were also taken but they all were negative.^63^ In the second case, the positive breast milk sample was tested 58 days after she was discharged from the Ebola treatment center.

All of the negative breast milk samples (*n* = 3) belong to mothers with positive EBOV infection; one EBOV‐positive mother was confirmed by RT‐PCR analysis of urine and two by antibody assessment.^57^, ^60^, ^62^ One positive breast milk sample belonged to a mother with negative EBOV serum status confirmed by RT‐PCR.^61^

In the studies that reported infant outcomes, children were aged from 0 days to 13 months. In one study with a mother of twins,^58^ one of the infants died before EBOV assessment and thus, the outcomes from the diseased infant were not included in the analysis. A total of seven infants were reported to be breastfed. However, only two of these reports provided information on breastfeeding practices. One study described a 6‐month‐old infant who was “almost exclusively” breastfed since birth.^57^ The second study reported on a 4‐month‐old infant who was exclusively breastfed since birth and was exposed to putative EBOV contaminated breast milk for at least 6 days after maternal disease onset.^58^ Among the five infants who were breastfed with laboratory‐confirmed EBOV in breast milk, one remained healthy^58^ and four died of EBOV infection.^16^, ^59^, ^61^ Only two infants were reported to be breastfed with EBOV‐negative breast milk, one infant died of laboratory‐confirmed EBOV^57^ and the second infant remained healthy with no signs or symptoms of infection.^62^

Two studies conducted genomic analysis and phylogenetic tracing of the virus isolated from the mother's breast milk and the child.^59^, ^61^ In both instances, the EBOV found in breast milk was correlated to the viral RNA isolated from the infant. A brief description of all included case reports and a table summarizing the results (Tables S1–3, online only).

### Certainty of evidence

For the assessment of the overall certainty of the evidence, we employed the GRADE approach against the retrieved data. For observational studies, like the ones included in this analysis, the GRADE approach starts the rating for the certainty of evidence as low certainty. The certainty of the evidence was further downgraded to very low certainty owing to a high risk of bias since there were few breast milk samples, no control groups, no follow‐up time, and there was scarce control of other possible confounders. The included studies also had a high risk of imprecision associated with a limited number of events reported for the outcomes of interest: (1) detection of EBOV in breast milk samples from women with suspected or confirmed infection and (2) EBOV infection of infants breastfeeding from a woman with confirmed or suspected EBOV infection. With only eight reports included, further research is needed to have greater confidence in the estimated effects of the studied outcomes.

## Discussion

### Principal findings

Breast milk is the first feeding of choice for infants not only for its nutritional value and protective roles but also for the overall health benefits for both mother and child.^68^, ^69^, ^70^, ^71^ However, the transmission of infectious pathogens through breast milk has been documented in the literature and, in the cases of cytomegalovirus, human T‐lymphotropic virus I (HTLV‐I), or HIV‐infected mothers, breastfeeding is linked to viral transmission.^72^, ^73^, ^74^ To safeguard the health of the child, and whenever feasible alternatives to breast milk are available, avoidance of breastfeeding practices is recommended as a control and management of vertical transmission.^71^, ^75^, ^76^ In the context of EBOV, it remains unclear whether breast milk is the main source of mother‐to‐child transmission. In light of the evidence, it appears that current recommendations merit no change. The child should be separated from the breastfeeding woman and infants younger than 6 months of age should be provided with a breast milk substitute that is acceptable, feasible, affordable, sustainable, and safe.^77^, ^78^

In our systematic search, we found limited evidence within an increasing number of reports following the recent and current EBOV outbreaks. Our analysis included a total of eight studies in which seven out of 10 breast milk samples, 70%, from different participants were positive for EBOV.^16^, ^58^, ^59^, ^61^, ^63^ We also reported on three independent studies that described four out of five children, 80%, who ingested EBOV‐positive breast milk and died of EBOV disease.^16^, ^59^, ^61^ At present, there are limited data available on the prevalence of EBOV in breast milk. Viral RNA in breast milk has been recorded from both symptomatic and asymptomatic women as early as 7 days^16^ up to 500 days after symptom onset.^63^ This suggests that in some instances, the virus can be partially cleared in blood, but persists in mammary tissue. Given that previous reports have shown that EBOV has a long‐term presence in human body tissues (e.g., 1–2 years in semen),^79^ there is a need to further define the mechanisms of EBOV excretion and prevalence in breast milk. Taken together, the evidence above may suggest that EBOV RNA shedding through breast milk can lead to, or might increase, the risk of mother‐to‐child transmission. However, the present data are not sufficient to prove either maternal‐to‐child viral transmission or infectivity of breast milk samples. This is mainly due to the very low certainty of the evidence, a limited number of reports, and the lack of simultaneously evaluating other potential modes of transmission.

The majority of the reports that evaluate maternal secretions as plausible modes of EBOV transmission do so by detecting the presence of RNA within a sample without simultaneously evaluating other potential transmission routes and mostly employing molecular‐based testing tools. Molecular‐based assays, including RT‐PCR, might be preferred because they are more sensitive than antibody‐ or antigen‐based tools; however, their sensitivity is reduced once the patient enters the convalescence phase of EBOV disease (∼13–45 days after disease onset).^80^ Additionally, as noted by the authors of one study in our review,^63^ commercially available RT‐PCR assays can have different sensitivities.^81^ Another limiting factor of exclusively utilizing molecular testing to confirm specimens is that they are designed only to identify the presence or absence of the pathogen's genetic material. To determine whether the isolated viral particles are infective as well as to establish clear epidemiological links with maternal viral isolates, other techniques that include viral culturing and RNA sequencing are required. We only found two studies that conducted genotypic and phylogenetic analyses of specimens to trace and correlate the virus isolated from the child to the one found in maternal breast milk samples.^59^, ^61^ This is possibly due to the many requirements to carry out such assays, which include, but are not limited to, having access to laboratory facilities with specialized equipment and trained personal to process EBOV samples.

Serological assays to determine the presence of anti‐EBOV antibodies should also be performed to complement RT‐PCR results in maternal and child cohorts. The identification of anti‐EBOV IgM or IgG might provide useful information regarding both maternal and infant exposure to EBOV as well as to facilitate the differentiation of acute and convalescent phases of the disease, especially when mothers are asymptomatic. We found three studies that assessed maternal antibodies^59^, ^60^, ^62^ and one study that characterized antibodies from an infant.^58^ One out of two studies that analyzed breast milk samples identified anti‐EBOV IgG antibodies.^60^, ^62^ The meaning of these changes in humoral responses and their potential association with viral clearance, passive immunity from mother to child, and safety to breastfeed remains largely unknown.

A systematic review on the effects of EBOV disease on pregnant and breastfeeding women^82^ showed mortality estimates on the basis of a total of 52 articles reporting on 274 pregnant women with EBOV disease. These estimates, accounting for past and recent outbreaks of *Zaire ebolavirus*, were determined to be 72% among pregnant women. Although these rates are similar to those found in the general population, they remain relatively higher than the WHO's estimated average for EBOV disease fatality rates (50%).^4^ Estimates of pregnancy outcomes when a woman is infected with EBOV were also included. Only 12% of the pregnancies were found to result in live‐born neonates, whereas adverse pregnancy outcomes, such as fetal loss and maternal death, were most abundant and estimated to have rates of 52% and 33%, respectively. Similar to our analysis, the authors also reported the presence of EBOV particles within maternal bodily fluids and tissues—amniotic fluid, placenta, fetal tissue, vaginal secretions, menstrual blood, and breast milk—highlighting the diverse modes of viral excretion and potential routes of vertical transmission. Our review here confirms and expands on these observations by including a detailed description of the articles and assessing additional reports with laboratory‐confirmed breast milk samples.

### Current infant feeding recommendations

Considering the high risk of morbidity and mortality of EBOV disease, we concur with previous reports and current WHO EBOV breastfeeding guidelines,^55^, ^77^ in that breastfeeding should be ceased when a woman is suspected or has been confirmed of EBOV disease. When caring for infants who are younger than 6 months, the guidelines state that whenever alternative feeding options are available, provide a breast milk substitute, such as infant formula or cow's milk. Breastfed children aged 6 months to 2 years should also be fed with milk substitutes along with complementary feeding. Moreover, it is recognized that the cessation of breastfeeding might not be feasible, accepted, affordable, or considered safe and, thus, this recommendation should be evaluated on a case‐by‐case basis. In instances where access to potable water or milk substitutes are not available, breastfeeding guidelines suggest continuing with breastfeeding practices but under close monitoring for early detection of signs and symptoms of EBOV in the infant.

Because of the nutritional and immunological benefits of breast milk, an EBOV‐positive mother might opt to breastfeed even when aware of the potential risks of transmission. Although not recommended, this choice should be respected and supported by healthcare workers without stigmatization and with clear individual counseling. In such instances, pasteurization of breast milk samples might be a potential alternative to continuing breastfeeding while also reducing the risks associated with EBOV‐positive breast milk. Previous reports in which breast milk samples were inoculated with infective EBOV particles suggest that the particles can be neutralized after undergoing pasteurization.^83^ While these results seem promising, we found no additional studies or specific recommendations on the effectiveness of pasteurization of EBOV‐contaminated breast milk samples. For general pasteurization of human breast milk samples, for both microbiological safety and to preserve the nutritional and immunological quality of the breast milk, human breast milk banking guidelines suggest Holder pasteurization by heating the breast milk at 62.5 °C for 30 minutes.^84^ However, it remains to be determined if pasteurization can be effectively and safely implemented in settings of high EBOV incidence.

### Study limitations

Although our analysis of reports that included laboratory‐confirmed breast milk specimens provided additional evidence suggesting that breast milk intake might be a source of mother‐to‐child EBOV transmission, we acknowledge that the certainty of the evidence is very low. Additionally, the present analysis was limited by the number of studies, the type of study design, and the lack of ability to exclude other probable means of transmission. As a result, we cannot confirm nor refute that the detection of viral RNA or the presence of viral particles within breast milk is associated with mother‐to‐child transmission.

### Implications for future research

As we move toward increasing our understanding of maternal‐to‐child modes of transmission, future studies should focus on conducting simultaneous in‐depth characterization of maternal bodily fluids to eliminate other potential routes of transmission. This includes performing additional assays that help clarify if the viral particles found in breast milk can cause infection and correlate the viral loads in breast milk with the infant's clinical outcomes. Implementing viral sequencing and antibody testing, where feasible, may also offer new insights into mother‐to‐child transmission and immunity.

## Author contributions

S.M., J.L.F., P.R.S., J.P.P.R., M.N.G.C., and L.R. conceptualized the systematic review. S.M., J.L.F., E.C.T., M.M.R., M.N.G.C., L.R., M.P.Z., J.C., and J.A. helped design the search strategy protocol. E.C.T. and K.G.K. updated and carried out the search strategy. M.M.R., E.C.T., P.R., S.S.M., J.A., A.J.L., J.C., and M.P.Z. participated in study screening. M.M.R. and E.C.T. extracted the data from the articles that met the inclusion criteria and wrote the manuscript. M.M.R. and E.C.T. contributed equally to this work. All authors reviewed and provided feedback on the manuscript.

## Disclaimer

The authors alone are responsible for the views expressed in this systematic review, and they do not necessarily represent the views, decisions, or policies of the institutions with which they are affiliated.

## Competing interests

The authors declare no competing interests relevant to this review. In the interest of full disclosure, S.M. has an equity interest in a startup commercializing some of his research focused on point‐of‐care assays for determining micronutrient status.

## Supporting information


**Table S1**. Summary of maternal outcomes.
**Table S2**. Summary of infant outcomes.
**Table S3**. Summary of the laboratory assessment of breast milk samples.
