# Supplementary material for: Presence of Ebola virus in breast milk and risk of mother‐to‐child transmission: synthesis of evidence
Source: Ann N Y Acad Sci. 2020 Oct 28;1488(1):33–43. doi: 10.1111/nyas.14519 (PMC8048832; doi:10.1111/nyas.14519)
Supplement: Supplementary file 1 — Table S1. Summary of maternal outcomes. Table S2. Summary of infant outcomes. Table S3. Summary of the laboratory assessment of breast milk samples. [file NYAS-1488-33-s001.docx]

# Supporting materials

## Search strategy

**Ebola virus transmission through breast milk**

July 21, 2020

Search strategy developed & performed by Kate Ghezzi-Kopel

Adapted from the search strategy developed by Joanne Abbott published as supplementary material in:

Colt, S., Garcia-Casal, M. N., Peña-Rosas, J. P., Finkelstein, J. L., Rayco-Solon, P., Weise Prinzo, Z. C., & Mehta, S. (2017). Transmission of Zika virus through breast milk and other breastfeeding-related bodily-fluids: A systematic review. *PLOS Neglected Tropical Diseases*, *11*(4), e0005528. <https://doi.org/10.1371/journal.pntd.0005528>

**MEDLINE (PubMed)**

1. Hemorrhagic Fever, Ebola[Mesh] OR Ebolavirus[Mesh] OR Ebola*[tiab]
2. (Sweat[MeSH] OR Blood[MeSH] OR Mucus[MeSH] OR Saliva[MeSH] OR Tears [MeSH] OR Infectious Disease Transmission, Vertical[MeSH] OR sweat*[tiab] OR blood*[tiab] OR mucus[tiab] OR serum[tiab] OR sera[tiab] OR fluid*[tiab] OR saliva[tiab] OR tears[tiab] OR vertical transmission[tiab] OR postnatal transmission[tiab] OR post natal transmission[tiab] OR maternal-infant transmission[tiab] OR adult-to-child[tiab] OR maternal-to-child[tiab] OR mother-to-child[tiab] OR MTCT[tiab] OR PMTCT[tiab])
3. (Breast Feeding[Mesh] OR Milk, Human[Mesh] OR Lactation[Mesh] OR colostrum[mesh] OR Breastfe*[tiab] OR breast fe*[tiab] OR breastmilk[tiab] OR breast milk[tiab] OR human milk[tiab] OR maternal milk[tiab] OR prelacteal feed*[tiab] OR lactati*[tiab] OR colostrum[tiab] OR mixed feeding[tiab] OR mother’s milk[tiab] OR expressed milk[tiab] OR milk bank*[tiab])
4. (Pregnant Women [Mesh] OR Pregnancy [Mesh] OR Prenatal Care [Mesh] OR Mothers[Mesh] OR infant[MeSH] OR pregnan*[tiab] OR gestat*[tiab] OR perinatal[tiab] OR antenatal[tiab] OR parturi*[tiab] OR mother*[tiab] OR infant*[tiab] OR maternal[tiab] OR baby[tiab] OR babies[tiab] OR newborn*[tiab] OR neonat*[tiab])
5. #4 AND #2
6. #3 AND #1
7. #5 AND #1
8. #6 OR #7

**Cochrane Library (Cochrane)**

1. MeSH descriptor: [Hemorrhagic Fever, Ebola] explode all trees
2. MeSH descriptor: [Ebolavirus] explode all trees
3. (Ebola*):ti,ab,kw
4. #1 OR #2 OR #3
5. MeSH descriptor: [sweating] explode all trees
6. MeSH descriptor: [blood] explode all trees
7. MeSH descriptor: [mucus] explode all trees
8. MeSH descriptor: [saliva] explode all trees
9. MeSH descriptor: [tears] explode all trees
10. MeSH descriptor: [Infectious Disease Transmission, Vertical] explode all trees
11. ("sweat*" OR "blood*" OR "mucus" OR "serum" OR "sera" OR "fluid*" OR "saliva" OR "tears" OR "vertical transmission" OR "postnatal transmission" OR "post natal transmission" OR "maternal-infant transmission" OR "adult-to-child" OR "maternal-to-child" OR "mother-to-child" OR "MTCT" OR "PMTCT")
12. #5 OR #6 OR #7 OR #8 OR #10 OR #11
13. MeSH descriptor: [Breast Feeding] explode all trees
14. MeSH descriptor: [Milk, Human] explode all trees
15. MeSH descriptor: [Lactation] explode all trees
16. MeSH descriptor: [Colostrum] explode all trees
17. ("Breastfe*" OR "breast fe*" OR "breastmilk" OR "breast milk" OR "human milk" OR "maternal milk" OR "prelacteal feed*" OR "lactati*" OR "colostrum" OR "mixed feeding" OR "**mother’s** milk" OR "expressed milk" OR "milk bank*"):ti,ab,kw
18. #12 OR #13 OR #14 OR #15 OR #16
19. MeSH descriptor: [Pregnant Women] explode all trees
20. MeSH descriptor: [Pregnancy] explode all trees
21. MeSH descriptor: [Prenatal Care] explode all trees
22. MeSH descriptor: [Mothers] explode all trees
23. MeSH descriptor: [Infants] explode all trees
24. ("pregnan*" OR "gestat*" OR "perinatal" OR "antenatal" OR "parturi*" OR "mother*" OR "infant*" OR "maternal" OR "baby" OR "babies" OR "newborn*" OR "neonat*"):ti,ab,kw
25. #19 OR #20 OR #21 OR #22 OR #23 OR #24
26. #25 AND #12
27. #18 AND #4
28. #26 AND #4
29. #27 OR #28

**Embase (Ovid)**

1. Ebola hemorrhagic fever/
2. (ebola*).mp
3. #1 OR #2
4. Sweat/ OR Blood/ OR Mucus/OR Saliva/ OR lacrimal fluid/
5. (sweat* OR blood* OR mucus OR serum OR sera OR fluid* OR saliva OR tears OR vertical transmission OR postnatal transmission OR post natal transmission OR maternal-infant transmission OR adult-to-child OR maternal-to-child OR mother-to-child OR MTCT OR PMTCT).mp
6. #4 OR #5
7. Exp breast feeding/ OR exp breast milk/ OR lactation/ OR exp colostrum/
8. (Breastfe* OR breast fe* OR breastmilk OR breast milk OR human milk OR maternal milk OR prelacteal feed* OR lactati* OR colostrum OR mixed feeding OR mother's milk OR expressed milk OR milk bank*).mp
9. #7 OR #8
10. Exp Pregnant woman/ OR exp pregnancy/ OR exp prenatal care/ OR exp mother/ OR exp infant
11. (pregnan* OR gestat* OR perinatal OR antenatal OR parturi* OR mother* OR infant* OR maternal OR baby OR babies OR newborn* OR neonat*).mp
12. #10 OR #11
13. #12 AND #6
14. #9 AND #3
15. #13 AND #3
16. #14 OR #15

**CINAHL (Ebsco)**

1. MH("Hemorrhagic Fever, Ebola" OR "Ebola Virus")
2. TX(ebola*)
3. S1 OR S2
4. MH(Sweat OR Blood+ OR Mucus OR Saliva OR Tears OR "Disease Transmission, Vertical")
5. TX(sweat* OR blood* OR mucus OR serum OR sera OR fluid* OR saliva OR tears OR "vertical transmission" OR "postnatal transmission" OR "post natal transmission" OR "maternal-infant transmission" OR "adult-to-child" OR "maternal-to-child" OR "mother-to-child" OR "MTCT" OR "PMTCT")
6. S4 OR S5
7. MH("Breast Feeding+" OR "Milk, Human+" OR Lactation OR Colostrum)
8. TX(Breastfe* OR "breast fe*" OR breastmilk OR "breast milk" OR "human milk" OR "maternal milk" OR "prelacteal feed*" OR lactati* OR colostrum OR "mixed feeding" OR "mother's milk" OR "expressed milk" OR "milk bank*")
9. S7 OR S8
10. MH(" Pregnant woman" OR "pregnancy" OR "prenatal care" OR "mother" OR "infant")
11. TX(pregnan* OR gestat* OR perinatal OR antenatal OR parturi* OR mother* OR infant* OR maternal OR baby OR babies OR newborn* OR neonat*)
12. S10 OR S11
13. S12 AND S6
14. S9 AND S3
15. S13 AND S3
16. S14 OR S15

**Web of Science Core Collection (Clarivate Analytics) / Biosis / Scielo**

1. TS=("ebola*")
2. TS=("sweat*" OR "blood*" OR "mucus" OR "serum" OR "sera" OR "fluid*" OR "saliva" OR "tears" OR "vertical transmission" OR "postnatal transmission" OR "post natal transmission" OR "maternal-infant transmission" OR "adult-to-child" OR "maternal-to-child" OR "mother-to-child" OR "MTCT" OR "PMTCT")
3. TS=("Breastfe*" OR "breast fe*" OR "breastmilk" OR "breast milk" OR "human milk" OR "maternal milk" OR "prelacteal feed*" OR "lactati*" OR "colostrum" OR "mixed feeding" OR "mother’s milk" OR "expressed milk" OR "milk bank*")
4. TS=("pregnan*" OR "gestat*" OR "perinatal" OR "antenatal" OR "parturi*" OR "mother*" OR "infant*" OR "maternal" OR "baby" OR "babies" OR "newborn*" OR "neonat*")
5. #4 AND #2
6. #3 AND #1
7. #5 AND #1
8. #6 OR #7

**Global Index Medicus** <https://pesquisa.bvsalud.org/gim/advanced/?lang=en>

1. ("ebola*") AND ("Breastfe*" OR "breastmilk" OR "breast milk" OR "human milk" OR "maternal milk" OR "prelacteal feed*" OR "lactati*" OR "colostrum" OR "mixed feeding" OR "mother’s milk" OR "expressed milk" OR "milk bank*" OR "sweat*" OR "blood*" OR "mucus" OR "serum" OR "sera" OR "fluid*" OR "saliva" OR "tears" OR "vertical transmission" OR "postnatal transmission" OR "post natal transmission" OR "maternal-infant transmission" OR "adult-to-child" OR "maternal-to-child" OR "mother-to-child" OR "MTCT" OR "PMTCT")

**Native health research database** [**https://hslic-nhd.health.unm.edu/Home/**](https://hslic-nhd.health.unm.edu/Home/)

Search term: ebola

## Summary of case reports:

### Study 1: Cases 1 and 2

Two case reports that occurred at Gulu Regional Hospital during an outbreak of Ebola hemorrhagic fever in Gulu, Uganda ^1^. Breast milk samples were collected from two patients at days 7 and 15 after disease onset (Case 1 and 2, respectively). Case 1 was classified as acute phase due to concurrent evaluation of serum samples via ELISA and/or RT-PCR resulted positive for EBOV. The patient in Case 2 was classified as convalescent given that previous serum EBOV positive results reverted to negative and/or showed the presence of IgG via ELISA specific antigen test. In both cases, the children of the infected mothers were reported to be breastfed and they both died of laboratory-confirmed Ebola hemorrhagic fever.

### Study 2: Cases 3

Moreau and collaborators describe two mother-child pairs during the mists of the 2014 EBOV outbreak in Guinea ^2^. However, only the breast milk from one mother-and-child pair was tested for EBOV and thus, the second case is briefly described but not included in our analysis. The first mother-child pair (Case 3), the mother, a woman in her late 30s presented symptoms of acute EBOV disease that included high fever, intense fatigue, headache, muscle and abdominal pain, vomiting, and diarrhea. The woman was admitted to the Ebola Treatment Centre of Médecins Sans Frontières in Guéckédou. On day 14 after symptom onset, she tested negative for EBOV infection via RT-PCR blood test. On the same day, her almost exclusively breastfed six-month infant developed fever, diarrhea, and severe weakness. The infant also tested positive for EBOV infection. Sixteen days after maternal symptom onset, the mother’s blood and urine were assessed for EBOV via RT-PCR, the results were negative and positive, respectively. The following day, a sample of her breast milk was evaluated for the presence of EBOV RNA by RT-PCR. Since the results of the breast milk sample were negative, the breastfeeding practices that were ceased when the mother was admitted were restarted. Three days later, 6 days after symptom onset, the infant died of laboratory-confirmed EBOV infection.

This study also describes a woman in her mid-20s that 4 days after giving birth developed a high fever, severe weakness, myalgia, arthralgia, anorexia, dysphagia, hiccups, abdominal pain, diarrhea, and vaginal discharged. Six days after symptoms started, the woman was taken to the Ebola Treatment Centre where she tested positive to EBOV infection via RT-PCR blood test. Upon her admission, her breastfed 10-month-old infant was separated from the mother and breastfeeding practices were stopped. Although no breast milk samples were collected or tested for EBOV, six days later the child developed a fever and was tested for EBOV infection. The RT-PCR blood analysis was negative at 1 and 3 days after symptom onset. The infant was followed for 21 days, in which period no signs or symptoms of EBOV disease were observed.

### Study 3: Case 4

A woman and her exclusively breastfed 4-month old twins were reported as EBOV contacts during the 2015 EBOV outbreak in Guinea ^3^. Soon after, one of the twins (baby 1) died with clinical symptoms that resemble an EBOV infection. Baby 1 died and was buried without laboratory confirmation of EBOV disease. At 16 days after baby 1 died, the mother was presenting symptoms of headache, loss of appetite, abdominal pain, joint pain, dysphagia, conjunctival injection, and myalgia. One day after been admitted to an Ebola treatment center, the mother’s blood tested positive for EBOV by RT-PCR. Although breastfeeding of baby 2 was ceased upon the mother’s admission to the treatment center, the child was breastfed for at least 6 days while his mother was symptomatic. Breast milk samples were collected on days 6, 14, and 21 after her admission. Given than all three breast milk samples resulted positive for EBOV, the mother was given drugs to dry up her milk supply, and baby 2 was tested three times for EBOV infection (days 1, 4, and 23 after maternal admission). Baby 2 remained healthy during the follow-up period of 26 days, in which serological testing for the presence of EBOV-specific antibodies (IgM and IgG) remained negative.

### Study 4: Case 5

A study reported on a familial cluster infected with EBOV during the 2015 outbreak in Mamusa, Port Loko District of Sierra Leone ^4^. A woman, her daughter, and sister were presumably exposed to EBOV while assisting in the childbirth of a close relative. After her relative and newborn died of laboratory-confirmed EBOV infection, the woman, her 13-month-old breastfeeding daughter, and sister were put in quarantine. During the observational period, the woman developed red conjunctivae but was never tested for EBOV infection. Three days after their release, her daughter and sister developed signs and symptoms of EBOV disease. The woman’s daughter died 3 days after disease onset. A subsequent genomic and phylogenetic analysis of the EBOV isolated from both mother and child shared a common origin and thus, EBOV infection might have been vertically transmitted from mother to child during the quarantine period.

### Study 5: Case 6

Sissoko and others reported on a maternal-to-child transmission that occurred in Dubréka, Guinea ^5^. A 9-month-old infant presented symptoms of EBOV disease such as fever, diarrhea, vomiting, and cough. The child was taken to the University Hospital in Conakry where her condition worsened and, subsequently, she died of respiratory distress. A saliva specimen from the child tested positive for EBOV disease. Given that neither the child nor her parents were in contact with EBOV infected patients or survivors, the parents received the rVSV-ZEBOV vaccine. Seroconversion analysis of the parents showed the presence of EBOV-specific IgG but not IgM, suggesting prior asymptomatic EBOV infection. To further investigate other potential routes of transmission, other bodily fluid specimens that included blood, urine, breast milk, and semen from the parents was collected and analyzed by EBOV specific RT-PCR kit. Maternal breast milk and paternal semen tested positive for the presence of EBOV RNA. Genomic and phylogenetic analyzes of the virus isolated from this familial cluster demonstrated that the virus found in the breast milk appeared ancestral to the one found in the infant suggesting vertical transmission. Whereas the virus isolated from the semen was from the same lineage, but the paternal virus did not share a molecular link to the virus isolated from the mother and child.

### Study 6: Case 7

A study by Kamali and collaborators reported on the delivery of a woman who contracted EBOV infection ~4 months before becoming pregnant ^6^. The mother, a 29-year-old physician, was working in West Africa during the 2014 EBOV outbreak. After providing care to a patient with EBOV infection, she presented symptoms of arthralgia and myalgia and was later confirmed to be EBOV positive via RT-PCR. As the disease progressed, she also developed fever, vomiting, and diarrhea. She received palliative care and was discharged after spending 13 days in an Ebola Treatment Center. Twenty-two weeks after her last negative EBOV tests, the woman became pregnant. She traveled to California, USA, where she received prenatal care and delivered a healthy neonate at 40 weeks, 1 day of gestation. Before delivery, her blood was tested for EBOV. Although the results for RT-PCR were negative, antibodies were found (IgG 1 ≤ 1:1600, IgM negative). During delivery and after delivery maternal samples such as vaginal secretions, amniotic fluid, cord blood, placenta, umbilical cord, breast milk (collected at 16 hours after birth), and oral and ear swabs from the neonate were collected. All analyzed samples via RT-PCR were negative. Attempts to isolate the virus from breast milk, placenta, amniotic fluid, and cord blood samples were unsuccessful. Colostrum samples were analyzed by ELISA to detect IgM and IgG antibodies against EBOV’s antigens. No antibodies were found. At six weeks after delivery, both mother and child were doing well with no signs or symptoms of EBOV disease.

### Study 7: Case 8

A familial cluster of EBOV composed of mother, father, and their four children were identified in an outbreak surveillance effort in Liberia ^7^. The presumed index case was the couple’s older son, a 15-year-old boy (Patient A) presenting symptoms of fatigue, generalized myalgia, arthralgia, fever, diarrhea, and hematemesis. After the teenager was confirmed as an EBOV positive case via RT-PCR, the remaining household members were transferred to an Ebola treatment center for assessment. RNA from EBOV was identified in blood samples from the father (Patient B), 8-yeard-old boy (Patient C), but it was not detected in specimens from the mother (Patient D) or the couple’s two other sons (2- month-old infant [Patient E] and a 5-year-old boy[Patient F]). Genomic analysis from viral samples from the father and his two EBOV positive children showed a strong epidemiological link between these cases, an EBOV from LB5 sub-lineage that circulated in Liberia in 2015. Further analysis of blood specimens collected from the parents showed high titers of both IgM and IgG antibodies. Serological analysis of blood specimens from their 2-month-old son showed evidence of IgG antibodies, presumably transferred from consuming IgG positive maternal breast milk. Except for patient A who died six days after EBOV diagnosis, all other family members were reported alive and well up to six months after initial tests.

### Study 8: Case 9 and 10

Ebola RNA shedding was studied in a prospective, multi-center, open cohort of Ebola disease survivors after discharged from Ebola treatment centers in Guinea^8^. As part of the PostEbogui study, 168 breast milk samples belonging to 109 EBOV patients were assessed. Ebola RNA virus was detected in a total of two samples. One breast milk sample (Case 9) was part of a subset of 16 samples that were re-tested employing an Ebola Xpert assay (RT-PCR). The positive breast milk sample was collected at 58-days after being discharged from the Ebola treatment center. This patient had additional samples, but they were all collected 2 years after recovery and hence, were not tested. No infant outcomes were reported.

The second positive breast milk sample (Case 10) belonged to a subset of 54 samples that had not been previously tested. Another 5 samples were collected from the same patient, taken between 1 and 10 months later. These samples, however, were all negative. In this case, the woman became pregnant seven months after being discharged from the Ebola treatment center. The positive breast milk sample was collected one month after delivery (~500 days after being discharged from the Ebola treatment center). The presence of RNA was reported to clear up and did not result in infant infection. Both of the samples were tested twice to exclude laboratory error.

Supplementary Table 1. Summary of maternal outcomes

|  | **Study** | **Design** | **Country** | **Participants** | **Maternal EBOV infection** | **Details of laboratory assessment** | **Clinical symptoms** | **Disease stage at the time of breast milk collection** |
| --- | --- | --- | --- | --- | --- | --- | --- | --- |
| Case 1 | Bausch 2007 ^1^ | Outbreak surveillance | Uganda | 2 breastfeeding women | Yes | Reported laboratory confirmation by viral culture, ELISA or RT-PCR | Not reported | Acute |
| Case 2 |  |  |  |  | Yes | Reported laboratory confirmation by viral culture, ELISA or RT-PCR | Not reported | Convalescent |
| Case 3 | Moreau 2015^a^ ^2^ | Case report | Guinea | 2 mother and infant pairs | Yes | Blood RT-PCR was negative at 14 and 16 days after illness onset. Urine RT-PCR was positive at 16 days after illness onset. | High fever, intense fatigue, headache, muscle and abdominal pain, vomiting, and diarrhea. 16 days before breastmilk sample was tested | Convalescent |
|  |  |  |  |  | Yes | Blood RT-PCR positive 6 days after illness onset. | Febrile syndrome four days after given birth. Later, she developed a high fever, severe weakness, myalgia, arthralgia, anorexia, dysphagia, hiccups, abdominal pain, diarrhea, and bloody vaginal discharge. | Acute |
| Case 4 | Nordenstedt 2015^b 3^ | Case report | Guinea | 1 mother and twins | Yes | Blood RT-PCR was positive 17 days after baby 1's illness onset. | Headache, loss of appetite, abdominal pain, joint pain, dysphagia, conjunctival injection, and myalgia | Acute |
| Case 5 | Arias 2016 ^4^ | Outbreak surveillance-case report | Sierra Leone | 1 mother and infant pair | No | Blood RT-PCR was negative. | Mostly Asymptomatic (red conjunctivae) | Asymptomatic |
| Case 6 | Sissoko 2017 ^5^ | Case report | Guinea | 1 mother and infant pair | Yes | Blood RT-PCR was negative. Antibody assessment by ELISA showed IgM negative, IgG positive. | Asymptomatic | Unknown, the mother was asymptomatic |
| Case 7 | Kamali 2016 ^6^ | Case report | United States of America | 1 mother and child pair | Yes | Blood RT-PCR was positive (13 months before birth), but negative at birth. Anti-Ebola virus serum antibodies were detected by ELISA before birth: IgG >1:1600, IgM negative. | Arthralgia, myalgia, fever, vomit, and diarrhea. After recovery, the woman suffered from fatigue, anorexia, arthralgia, and alopecia. These symptoms lasted for 2-3 months. | Convalescent |
| Case 8 | Dokubo 2018^c 7^ | Outbreak surveillance-case report | Liberia | A familial cluster that included a couple and their four sons  (1 mother and child pair) | Yes | Blood RT-PCR was negative. Anti-Ebola virus serum antibodies were detected by ELISA: 1436 EU/mL IgM and 1,436 EU/mL IgG 851,334 | Asymptomatic | Convalescent |
| Case 9 | Keita 2019 ^8^ | Cohort, prospective, open, multi-center | Guinea | Description of two EBOV-positive breast milk samples collected in a cohort study of 109 EBOV survivors. | Yes, both women were recruited to the study after being discharged from an Ebola treatment center | Not described | Not reported | Convalescent |
|  |  |  |  |  |  | Not described | Not reported | Convalescent |
|  | ^a^ Only one out of the two reported cases included a breast milk sample. The case without breast milk sample is not included in the analysis.  ^b^ This study reports on a mother with twins. Unfortunately, one of the infants (baby 1) died before EBOV assessment and is not included in the analysis.  ^c^ Maternal serological status was assessed after immunization with vesicular stomatitis virus (rVSV)-based vaccine expressing the glycoprotein of the Zaire Ebola virus (rVSV-ZEBOV)  NA = Not available | | | | | | | |

Supplementary Table 2. Summary of infant outcomes

|  | **Study** | **Infant EBOV infection** | **Details of laboratory assessment** | **Viral RNA sequencing** | **Symptoms** | **Mortality** | **Child age** | **Was the child breastfed?** | **Description of breastfeeding practices** |
| --- | --- | --- | --- | --- | --- | --- | --- | --- | --- |
| Case 1 | Bausch 2007 ^1^ | Yes | Reported positive laboratory confirmation but no additional details were reported. | NA | Not reported | Yes | Not reported | Yes | Not reported |
| Case 2 |  | Yes | Reported positive laboratory confirmation but no additional details were reported. | NA | Not reported | Yes | Not reported | Yes | Not reported |
| Case 3 | Moreau 2015^a^ ^2^ | Yes | Blood RT-PCR positive | NA | Fever, diarrhea and severe weakness | Yes | 6 months | Yes | "Almost exclusively for 6 months" |
|  |  | No | Blood RT-PCR negative | NA | Fever | No | 10 days | Yes | "Child was breastfed since birth" |
|  | Nordenstedt 2015 ^b, c 3^ | Yes, suspected | Baby 1: Deceased and buried before maternal admission to the Ebola treatment center | NA | Fever | Yes | 4 months | Yes | Both children were exclusively breastfed during their first 4 months of life.  Baby 2 was breastfed for at least 6 days after maternal illness onset. |
| Case 4 |  | No | Baby 2: Blood RT-PCR negative at day 6 and 9 after maternal illness onset. Serological testing, performed on day 23, showed no signs of previous subclinical infection (ELISA, IgM, and IgG negative) | NA | None | No | 4 months | Yes |  |
| Case 5 | Arias 2016 ^4^ | Yes | Blood RT-PCR positive | Positive phylogenetical correlation of viral genome isolated in breast milk with the one isolated from the infant. | Not reported | Yes | 13 months | Yes | Not reported |
| Case 6 | Sissoko 2017 ^5^ | Yes | Buccal swap-Positive | Positive correlation with infant viral RNA with found virus found in breast milk samples | Fever, diarrhea, vomiting, and cough, severe vomiting, and diarrhea. Ultimately respiratory distress | Yes | 9 months | Yes | Not reported |
| Case 7 | Kamali 2016 ^6^ | No | Blood RT-PCR negative | No | No | No | 0 days of life | Not reported | Not reported |
| Case 8 | Dokubo 2018 ^7^ | Yes | Blood RT-PCR negative. Antibody serum analysis showed IgG positive (4524 EU/mL). IgM was not determined. | NA | No | No | 2 months | Yes | Not reported |
| Case 9 | Keita 2019 ^8^ | Yes | NA | NA | NA | NA | NA | NA | Not reported |
|  |  | Yes | NA | NA | Asymptomatic | NA | 1 month | NA | Not reported |
|  | ^a^ Only one out of the two reported cases includes the assessment of a breast milk sample. The case without breast milk samples is not included in the analysis.  ^b^ This study reports on a mother with twins. Unfortunately, one infant (baby 1) died before EBOV assessment and is not included in the analysis.  ^c^ Following Ebola virus-positive diagnosis the woman received cabergoline to cease the production of breast milk.  NA = Not available | | | | | | | | |

Supplementary Table 3. Summary of the laboratory assessment of breast milk samples

|  | **Study** | **RT-PCR** | **Culture** | **Viral RNA sequencing** | **Ebola Antibodies in Breast Milk** |
| --- | --- | --- | --- | --- | --- |
| Case 1 | Bausch 2007 ^1^ | Positive | Positive | NA | NA |
| Case 2 |  | Positive | Positive | NA | NA |
| Case 3 | Moreau 2015 ^2^ | Negative | NA | NA | NA |
|  |  | The breast milk sample was not assessed for the presence of EBOV. | | | |
| Case 4 | Nordenstedt 2015 ^3^ | Positive | NA | NA | NA |
| Case 5 | Arias 2016 ^4^ | Positive | NA | Positive correlation with the virus isolated from the child | NA |
| Case 6 | Sissoko 2017 ^5^ | Positive | NA | Positive correlation with the virus isolated from the child | NA |
| Case 7 | Kamali 2016 ^6^ | NA | Negative, no virus was recovered | No | No IgG or IgM antibodies were detected |
| Case 8 | Dokubo 2018 ^7^ | Negative | NA | NA | IgG antibodies were detected (827 EU/mL). IgM was not determined. |
| Case 9 | Keita 2019 ^8^ | Positive | NA | NA | NA |
|  |  | Positive | NA | NA | NA |
| NA = Not available | | | | | |
